# Does psychological treatment of major depression reduce cardiac risk biomarkers? An exploratory randomized controlled trial

**DOI:** 10.1017/S0033291722000447

**Published:** 2023-06

**Authors:** Frank Euteneuer, Marie Neuert, Stefan Salzmann, Susanne Fischer, Ulrike Ehlert, Winfried Rief

**Affiliations:** 1 Clinical Psychology and Psychotherapy, Department of Psychology, Medical School Berlin, Berlin, Germany; 2 Division of Clinical Psychology and Psychotherapy, University of Marburg, Marburg, Germany; 3 Clinical Psychology and Psychotherapy, Institute of Psychology, University of Zurich, Zurich, Switzerland

**Keywords:** Blood pressure, Cognitive behavioral therapy, heart rate variability, inflammation, major depression

## Abstract

**Background:**

Depression is associated with an increased risk for cardiovascular disease (CVD). Biological cardiac risk factors are already elevated in depressed patients without existing CVD. The purpose of this exploratory trial was to examine whether treating Major Depression (MD) with cognitive behavioral therapy (CBT) is associated with improvements in cardiac risk biomarkers and whether depressive symptom severity at baseline moderates treatment effects.

**Methods:**

Eighty antidepressant-free patients with MD were randomly assigned to CBT or waiting list (WL). Biological outcomes included long-term recordings (24-h, daytime, nighttime) of heart rate, heart rate variability (HRV), and blood pressure, as well as inflammatory markers such as C-reactive protein (CRP), interleukin (IL)-6, and tumor necrosis factor (TNF)-*α*. A sample of 40 age- and sex-matched non-clinical controls was also involved to verify biological alterations in MD at study entry.

**Results:**

Compared to WL, CBT was associated with a significant increase in overall HRV, as indexed by the 24-h and daytime HRV triangular index, as well as trend improvements in 24-h low-frequency HRV and daytime systolic blood pressure. Self-rated depressive symptom severity moderated (or tended to moderate) improvements in CBT for 24-h and daytime heart rate and several indices of HRV (especially daytime measures). Inflammatory treatment effects were not observed.

**Conclusions:**

CBT increased overall HRV in patients with MD. Initially more depressed patients showed the most pronounced cardiovascular improvements through CBT. These exploratory findings may provide new insights into the biological effects of psychological treatment against depression and must be confirmed through future research.

## Introduction

Depression is associated with an increased risk for the development of cardiovascular disease (CVD) (Gan et al., 2014; Harshfield et al., 2020; Li et al., 2019; Wu & Kling, 2016) and future cardiovascular events in patients with existing CVD (Carney & Freedland, 2017; Goldston & Baillie, 2008; Pelle, Gidron, Szabó, & Denollet, 2008; Spaderna et al., 2017). Cardiac risk biomarkers are already prominent in patients with depression in the absence of CVD. These biological factors include autonomic dysregulation such as reduced heart rate variability (HRV) and increased heart rate (Kemp et al., 2010; Koch, Wilhelm, Salzmann, Rief, & Euteneuer, 2019; Lake et al., 1982; Lehofer et al., 1997), and possibly elevated blood pressure (Ginty, Carroll, Roseboom, Phillips, & de Rooij, 2013; Meng, Chen, Yang, Zheng, & Hui, 2012). In addition to autonomic dysregulation, depression is associated with elevated circulating levels of inflammatory immune markers such as interleukin (IL)-6, tumor necrosis factor (TNF)-*α* and C-reactive protein (CRP) (Dowlati et al., 2010; Howren, Lamkin, & Suls, 2009; Osimo et al., 2020). Based on these findings, it has been suggested that autonomic dysregulation and inflammation may partially mediate the link between depression and CVD (Musselman, Evans, & Nemeroff, 1998; Nicholson, Kuper, & Hemingway, 2006; Shaffer & Ginsberg, 2017; Sgoifo, Carnevali, de los Angeles Pico Alfonso, & Amore, 2015; Williams & Steptoe, 2007).

Previous studies suggest bidirectional associations of depression with autonomic and immunological alterations (Copeland, Shanahan, Worthman, Angold, & Costello, 2012; Deverts et al., 2010; Huang et al., 2018; 2019; Matthews et al., 2010; Valkanova, Ebmeier, & Allan, 2013). Cognitive behavioral therapy (CBT) is the most-studied form of psychotherapy for MD (Cuijpers et al., 2013; Cuijpers, Cristea, Karyotaki, Reijnders, & Huibers, 2016; David, Cristea, & Hofmann, 2018). By changing dysfunctional thoughts and behaviors, CBT may have the potential to reduce cardiac risk biomarkers via multiple stress-related and behavioral mechanisms. These potential mechanisms include factors which (i) are targeted by CBT and (ii) may interact with autonomic dysregulation and inflammation, such as negative mood, perceived stress and emotional stress reactivity, poor cognitive and behavioral skills to cope with stressors, social withdrawal, as well as inactivity (Allen, Kennedy, Cryan, Dinan, & Clarke, 2014; Audet, McQuaid, Merali, & Anisman, 2014; Eller, Kristiansen, & Hansen, 2011; Gerteis & Schwerdtfeger, 2016; Kemp, Koenig, & Thayer, 2017; Kiecolt-Glaser, Derry, & Fagundes, 2015; Lee & Way, 2019; Plaisance & Grandjean, 2006; Sin, Sloan, McKinley, & Almeida, 2016; Uchino et al., 2018; Yang, Schorpp, & Harris, 2014).

Although considered the gold-standard for the psychological treatment of MD, one important and understudied question is, however, whether CBT reduces cardiac risk biomarkers in populations with MD without CVD. In terms of HRV, two randomized controlled trials (RCT) indicated that short CBT interventions (i.e. 4–6 weeks) combined with HVR biofeedback (Caldwell & Steffen, 2018) or breathing exercises (Chien, Chung, Yeh, & Lee, 2015) may improve specific measures of HRV. Both studies have important limitations, such as a small sample size of younger females (Caldwell & Steffen, 2018), a short treatment duration (Caldwell & Steffen, 2018; Chien et al., 2015), the inclusion of patients with antidepressant medication (Chien et al., 2015), as well as the use of short-term measures of HRV (Caldwell & Steffen, 2018; Chien et al., 2015) instead of long-term HRV recordings which are more stable, less affected by placebo effects and more predictive for cardiovascular events in populations without known CVD (Hillebrand et al., 2013; Task Force of The European Society of Cardiology and The North American & Society of Pacing and Electrophysiology, 1996). In terms of inflammation, one previous RCT suggests that CBT lowers IL-6 and TNF-*α* in a sample of young adults (Moreira et al., 2015), while two trials did not observe an overall effect of CBT on inflammatory markers (Euteneuer et al., 2017; Taylor et al., 2009).

The present RCT examined whether established CBT for depression is accompanied by reductions in cardiac risk biomarkers in patients with MD without CVD. We aimed to extend previous research in several regards. First, we exploratory studied biological effects of CBT for a wide range of measures with predictive value for CVD, such as heart rate (Greenland et al., 1999; Kannel, Kannel, Paffenbarger, & Cupples, 1987), indices of HRV (Hillebrand et al., 2013), blood pressure (Lewington, Clarke, Qizilbash, Peto, & Rory, 2002), as well as inflammatory markers such as CRP, IL-6 and TNF-*α* (Avan et al., 2018; Pearson, 2003; Subirana et al., 2018). Second, this RCT only included patients without antidepressant medication. This point is important because antidepressants, in particular tricyclic antidepressants, may reduce HRV (Kemp et al., 2016, 2010; Kemp, Quintana, & Malhi, 2011; Yeh et al., 2016). Third, we used 24-h long-term recordings for cardiac measures instead of short-term recordings. Long-term recordings are more predictive for CVD (Hillebrand et al., 2013) and provide the possibility to analyze geometric indices of HRV such as the triangular index, which has been suggested for prospective research because of the highest reliability among all HRV indices (Ziegler, Piolot, Strassburger, Lambeck, & Dannehl, 1999). In addition, long-term recordings enable the possibility to consider circadian aspects of cardiac measures (Bilan, Witczak, Palusiński, Myśliński, & Hanzlik, 2005; Bonnemeier et al., 2003; Carney et al., 2000; Li et al., 2011; Ohkubo et al., 2002; Parati et al., 2014). Based on a pre-post study suggesting that CBT improved heart rate and daytime HRV in severely depressed patients but not in mildly depressed patients with coronary heart disease (Carney et al., 2000), we examined whether the intensity of depressive symptoms at study entry moderates potential effects on biological outcomes. This trial should be considered exploratory, because a wide range of biological markers were assessed, aiming to identify potential biological responsiveness for future research.

## Materials and methods

This RCT was conducted from October 2015 to October 2019 in accordance with the World Medical Association Declaration of Helsinki and the ethical guidelines of the German Society for Psychology (see online Supplementary material for CONSORT 2010 checklist). The approval was given by the Institutional Review Board of the Department of Psychology at the University of Marburg (Approval number: 2014-26k). The study was funded by the German Research Foundation (DFG EU 154/2-1), and study registration took place (www.clinicaltrial.gov NCT 02787148).

### Participants

Eighty antidepressant-free patients with MD according to DSM-IV were (1:1) urn randomized to 14 weeks of CBT or a waitlist control condition (see online Supplementary material for recruiting, setting and intervention). A sample of 40 (1:2) age- and sex-matched non-clinical controls from the same community was also involved to verify potential baseline alterations in biological markers in MD.

### Depression assessment

Clinical diagnoses according to DSM-IV were verified using the SCID (Wittchen, Wunderlich, Gruschitz, & Zaudig, 1997). To verify the efficacy of the CBT intervention, the primary outcome was self-rated depressive symptom severity assessed with the Beck Depression Inventory (BDI)-II (Beck, Brown, & Steer, 1996; Hautzinger, Kühner, & Keller, 2006). Clinician-rated depressive symptom severity was also assessed using the Montgomery-Asberg Depression Rating Scale (MADRS) (Montgomery & Asberg, 1979; Schmidtke, Fleckenstein, Moises, & Beckmann, 1988). Clinical psychologists conducted clinical interviews and baseline MADRS assessments. MADRS assessment at the end of treatment was conducted by trained, supervised, and blinded research assistants.

### Inflammatory markers

Non-fasting blood samples were collected in EDTA-treated tubes (S-Monovette, Sarstedt, Nümbrecht, Germany) between 8:00 AM and 11:00 AM. Before each blood sampling, participants were queried about acute infections during the last 14 days, chronic infections, and illness. Plasma for CRP and cytokine measurements were separated by centrifugation at 2000 × ***g*** for 10 min at 4 °C, and plasma was stored at −80 °C until analysis. CRP and cytokines were analyzed using the V-PLEX Human CRP Kit and MSD cytokine assays (Meso Scale Discovery, Rockville, USA) according to the manufacturer's instructions. The sensitivity of the assays was 1.33 pg/ml for CRP, 0.04 pg/ml for TNF-*α*, and 0.06 pg/ml for IL-6.

### Ambulatory cardiovascular monitoring

Twenty-four-hour oscillometric blood pressure and electrocardiogram (ECG) monitoring for heart rate and HRV analyses was conducted during weekdays using a hybrid blood pressure/ECG monitor (card(X)plore, Meditech Ltd., Hungary). Participants were instructed to follow their typical daily activities but refrain from intense physical activity. Monitoring started between 8 AM and 11 AM. Considering circadian variations in heart rate, blood pressure, and HRV (Bilan et al., 2005; Bonnemeier et al., 2003; Li et al., 2011), these measures were calculated for the total 24-h recording and additionally for day- and nighttime. Using a fixed-narrow time interval approach, measures for day- and nighttime were calculated from standardized periods (i.e. daytime: 9 AM to 9 PM; nighttime: 1 AM to 6 AM) in which the retiring and rising periods (which are subject to considerable variation) were eliminated to increase reliability and comparability (Fagard, Brguljan, Thijs, & Staessen, 1996; O'Brien et al., 2013). CardioVisions 1.24 (Meditech Ltd., Hungary) was used to obtain parameters for heart rate, HRV, and blood pressure.

Heart rate was recorded continuously by a 3-channel Holter ECG at a sampling rate of 600 Hz using seven Ag/AgCl electrodes (Kendall H92SG, Cardinal Health Germany 507 GmbH, Germany) placed on the thoracic region. First, similar QRS complexes were automatically grouped and defined as normal or artifact beats. Next, QRS complexes were visually screened for incorrect beat detections and, in case of an incorrect reading, marked as artifact beat. All artifacts were excluded from the data. A minimum of 18 h of analyzable data and less than 5% artifacts of all analyzable beats were required for a recording to be accepted for further analyses (Crawford et al., 1999).

As recommended by common guidelines (Task Force of The European Society of Cardiology and The North American & Society of Pacing and Electrophysiology, 1996), the following time-domain HRV measures were assessed: the HRV triangular index and the standard deviation of all NN intervals (SDNN) for overall HRV, as well as the square root of the mean of the sum of the squares of differences between adjacent NN intervals (RMSSD) for estimating vagally mediated changes reflected in HRV. In terms of frequency-domain HRV, low-frequency (LF)-HRV (0.04–0.15 Hz) and high-frequency (HF)-HRV (0.15–0.4 Hz) were analyzed. HF-HRV reflects vagal modulation of HR. LF-HRV is more complex and may include both sympathetic and parasympathetic influences (Shaffer & Ginsberg, 2017; Task Force of The European Society of Cardiology and The North American & Society of Pacing and Electrophysiology, 1996). To determine LF and HF parameters, the software utilizes a Fast Fourier Transform (FFT) algorithm considering 4-min Hanning-windowed samples.

For ambulatory blood pressure monitoring (ABPM), the device was programmed to obtain blood pressure readings every 15 min during daytime (7 AM to 10 PM) and every 30 min during the night-time (10 PM to 7 AM). The inflatable cuff was placed on the participant's right arm. After the measurement, ABPM readings were manually screened for artifacts. As recommended, only strongly incorrect readings were deleted from recordings (O'Brien et al., 2003). Systolic blood pressure (SBP) values lower than 60 or higher than 260, and diastolic blood pressure (DBP) values lower than 40 and higher than 150 were defined as artificial readings and excluded. Daytime, nighttime and 24-h calculations based on weighted average blood pressure. Nocturnal SBP and DBP dipping was determined by subtracting the nighttime average blood pressure from the daytime average blood pressure. Thus, lower values on these different measures reflect lower nocturnal blood pressure dipping. This procedure yields adequate reliability for dipping values (Dimsdale et al., 2000).

### Data analysis

Baseline measures are reported as means with SDs for continuous variables and as numbers with percentages for categorical variables. Data distributions were inspected, and screening for extreme outliers was conducted (i.e. values more than three interquartile ranges above quartile 3). Potential differences in study variables between patients with MD and non-clinical controls were examined using Welch's *t* tests and χ^2^ tests. Constrained longitudinal data analysis (cLDA) with linear mixed models (Coffman, Edelman, & Woolson, 2016; Fitzmaurice, Laird, & Ware, 2011; Liang & Zeger, 2000; Liu, Lu, Mogg, Mallick, & Mehrotra, 2009) was applied to examine differences in changes from baseline to the end of treatment between both treatment groups. Constrained linear mixed models incorporated baseline adjustment (i.e. baseline means are constrained to be equal between the randomized groups;) with time (baseline, end of treatment) and time × treatment interactions as fixed factors and with a random intercept at patient level. Longitudinal data were analyzed on an intention-to-treat base using maximum likelihood estimation to account for missing data and dropouts, respectively. To examine moderating effects of depressive symptom severity, time × treatment × baseline severity interactions, as well as corresponding lower-order terms, were added to constrained linear mixed models. Following common suggestions for exploratory research (Althouse, 2016; Bender & Lange, 2001; Rothman, 1990; Rubin, 2017), we report and interpret results without adjusting for multiple testing. All *p*-values are two-tailed and analyses were carried out with SPSS version 20.0 for Windows (Chicago, SPSS, Inc.) and Mplus7 (Muthén & Muthén, 1998–2012).

## Results

### Baseline and trial characteristics

**Fig. 1.**
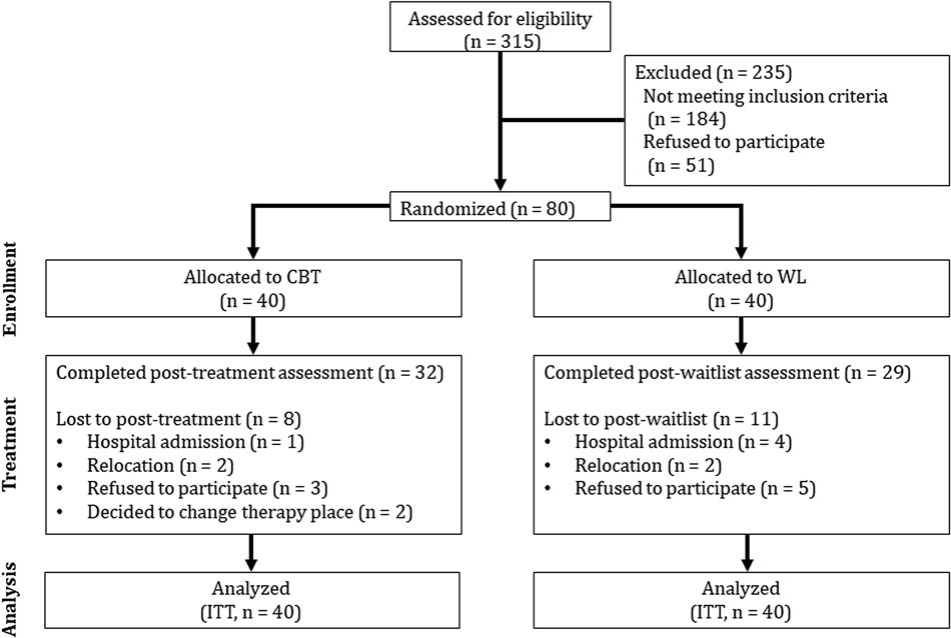
Flow of participants through each stage of the trial. CBT, cognitive-behavioral therapy; ITT, intention-to-treat; WL, waitlist.

A total of 80 patients with MD and 40 non-clinical controls participated in this study. As illustrated in the study flow (shown in Fig. 1), dropout rates from baseline to the end of treatment were 20% for the CBT group and 27.5% for the WL group. Missing values occurred, and extreme outliers in biological variables were also considered missing values. Extreme outliers were observed for the following variables: CRP (non-clinical controls: 0%, CBT group: 5.6%, WL group: 5.8%), IL-6 (non-clinical controls: 0%, CBT group: 2.8%, WL group: 1.4%), nighttime heart rate (non-clinical controls: 0%, CBT group: 1.4%, WL group: 0%), daytime HF-HRV (non-clinical controls: 0%, CBT group: 1.4%, WL group: 1.4%), daytime LF-HRV (non-clinical controls: 0%, CBT group: 0%, WL group: 1.4%), nighttime HF-HRV (non-clinical controls: 2.5%, CBT group: 0%, WL group: 1.4%), and nighttime SBP (non-clinical controls: 0%, CBT group: 0%, WL group: 1.4%). Among those who completed the study, overall analyzable data were available as follows: BDI-II (non-clinical controls: 100%, CBT group: 100%, WL group: 96%), MADRS (non-clinical controls: 95%, CBT group: 94%, WL group: 94%), CRP (non-clinical controls: 95.0%, CBT group: 87.5%, WL group: 78.3%), IL-6 (non-clinical controls: 95.0%, CBT group: 90.3%, WL group: 82.6%), TNF-*α* (non-clinical controls: 95.0%, CBT group: 93.1%, WL group: 84.1%), heart rate and HRV measures (non-clinical controls: 92.5–95.0%, CBT group: 80.6–86.1%, WL group: 76.8–81.2%), and blood pressure measures (non-clinical controls: 95.0%, CBT group: 80.6–93.1%, WL group: 76.8–84.1%). Fisher's exact test did not indicate significant differences for any study variable when comparing proportions of outliers (all *p*s ⩾ 0.489) or total missing data (all *p*s ⩾ 0.114) between both treatment groups.

Descriptive statistics for all groups and comparisons between patients with MD and non-clinical controls are presented in Table 1 (see online Supplementary material Table S1 for correlations between biological variables at study entry and Table S2 for correlations between changes in biological variables for each treatment group). Compared to non-clinical controls, patients with MD exhibited significantly higher levels in all inflammatory markers (i.e. CRP, IL-6 and TNF-*α*). Patients with MD showed significantly higher levels of 24-h and daytime heart rate, relative to non-clinical controls. With respect to HRV, patients had significantly lower levels of 24-h HRV triangular index, daytime HRV triangular index, daytime HF-HRV, daytime LF-HRV and daytime RMSSD, and a trend of reduced daytime SDNN. For blood pressure, higher levels of 24-SBP and daytime DBP, and a trend of higher daytime SBP and 24-h SBP, were observed in patients with MD compared to controls. There were no significant differences between groups for any other biological measures.

**Table 1.** Baseline .characteristics of patients with Major Depression and comparison of study variables with a nonclinical age- and sex-matched non-clinical control sample

Variable	Major depression			Non-clinical controls (*n* = 40)	Test statistics
	CBT group (*n* = 40)	WL group (*n* = 40)	Total (*n* = 80)		
Age	30.73 (10.02)	29.90 (10.27)	30.31 (10.09)	30.23 (9.63)	*t*(81.43) = 0.05, *p* = 0.963
Female, number, *n* (%)	26 (65.0)	22 (55.0)	48 (60.0)	24 (60.0)	χ^2^ (1) = 0.00, *p* = 1.000
Socioeconomic status^1^	8.89 (3.60)	8.31 (3.58)	8.60 (3.57)	10.00 (3.48)	*t*(79.57) = –2.01, *p* = 0.048
Body mass index, kg/m^2^	24.72 (4.36)	24.90 (4.70)	24.81 (4.50)	22.72 (2.03)	*t*(108.73) = 3.38, *p* = 0.001
Daily smoker, *n* (%)	16 (40.0)	17 (42.5)	33 (41.3)	5 (12.5)	χ^2^ (1) = 10.19, *p* = 0.001
Age of depression onset	21.53 (9.43)	22.05 (11.10)	21.78 (10.23)	N/A	N/A
Number of depressive episodes	4.90 (4.58)	4.05 (3.83)	4.50 (4.23)	N/A	N/A
Chronic depression (⩾2 years), *n* (%)	9 (22.5)	6 (15.0)	15 (18.8)	N/A	N/A
Depressive symptoms severity					
Self-rated, BDI-II	28.24 (8.70)	30.55 (8.07)	29.40 (8.42)	2.98 (2.84)	(107.77) = 25.33, *p* < 0.001
Clinician-rated, MADRS	28.48 (6.62)	29.58 (6.49)	29.04 (6.53)	3.08 (2.91)	*t*(114.53) = 29.73, *p* < 0.001
Immunological markers					
CRP, *μ*g/ml^2^	0.56 (0.58)	0.59 (0.59)	0.57 (0.58)	0.36 (0.33)	*t*(102.93) = 2.36, *p* = 0.020
IL-6, pg/ml	0.46 (0.27)	0.58 (0.28)	0.52 (0.28)	0.36 (0.20)	*t*(96.48) = 3.34, *p* = 0.001
TNF-*α*, pg/ml	1.09 (0.32)	1.04 (0.26)	1.07 (0.29)	0.96 (0.16)	*t*(106.97) = 2.55, *p* = 0.012
Heart rate					
24-h, beats/min	77.43 (11.11)	77.55 (7.18)	77.49 (9.32)	73.28 (6.58)	*t*(96.19) = 2.70, *p* = 0.008
Daytime, beats/min	84.80 (11.40)	85.85 (8.63)	85.33 (10.03)	79.01 (7.03)	*t*(96.89) = 3.81, *p* < 0.001
Nighttime, beats/min	63.59 (10.95)	64.18 (6.88)	63.89 (9.08)	63.22 (8.43)	*t*(78.92) = 0.38, *p* = 0.709
Heart rate variability					
Triangular index					
24-h	32.38 (8.79)	31.32 (7.15)	31.85 (7.97)	36.08 (9.92)	*t*(61.63) = −2.23, *p* = 0.029
Daytime	25.91 (7.35)	23.92 (5.74)	24.89 (6.60)	28.08 (7.35)	*t*(66.87) = –2.21, *p* = 0.030
Nighttime	20.44 (6.90)	19.47 (7.08)	19.96 (6.95)	22.30 (7.69)	*t*(67.92) = –1.54, *p* = 0.128
HF-HRV					
24-h, ms^2^	759.68 (547.51)	851.38 (720.08)	805.53 (636.53)	902.59 (766.94)	*t*(63.27) = –0.66, *p* = 0.514
Daytime, ms^2^	435.79 (282.00)	432.20 (360.52)	433.94 (322.40)	637.38 (500.05)	*t*(52.68) = –2.24, *p* = 0.030
Nighttime, ms^2^	1249.65 (1115.65)	1288.97 (1107.37)	1269.01 (1103.30)	1184.97 (1115.78)	*t*(73.66) = 0.37, *p* = 0.713
LF-HRV					
24-h, ms^2^	1401.85 (677.78)	1480.35 (799.32)	1441.10 (736.56)	1688.59 (927.21)	*t*(61.10) = –1.40, *p* = 0.166
Daytime, ms^2^	1213.91 (569.95)	1118.97 (492.50)	1165.75 (530.29)	1587.03 (868.30)	*t*(50.77) = –2.69, *p* = 0.010
Nighttime, ms^2^	1684.59 (966.56)	1706.41 (1083.42)	1965.50 (1019.03)	1821.11 (1152.38)	*t*(69.11) = –0.56, *p* = 0.577
RMSSD					
24-h, ms	39.24 (15.08)	40.50 (17.91)	39.87 (16.45)	42.41 (18.60)	*t*(66.65) = –0.70, *p* = 0.489
Daytime, ms	31.21 (12.12)	30.11 (13.34)	30.64 (12.68)	36.05 (13.48)	*t*(69.58) = –2.02, *p* = 0.048
Nighttime, ms	52.94 (26.54)	54.71 (29.10)	53.82 (27.65)	53.30 (29.28)	*t*(70.50) = 0.09, *p* = 0.929
SDNN					
24-h, ms	161.00 (40.38)	155.18 (34.25)	158.09 (37.27)	162.54 (40.46)	*t*(69.02) = –0.55, *p* = 0.582
Daytime, ms	121.12 (33.52)	113.42 (29.90)	117.16 (31.72)	129.22 (34.02)	*t*(69.08) = –1.79, *p* = 0.079
Nighttime, ms	114.03 (35.65)	108.21 (37.42)	111.12 (36.39)	124.35 (49.28)	*t*(57.80) = –1.44, *p* = 0.157
Blood pressure					
Systolic blood pressure					
24-h, mm Hg	119.16 (12.56)	123.72 (11.87)	121.41 (12.36)	117.32 (8.50)	*t*(100.59) = 2.05, *p* = 0.043
Daytime, mm Hg	124.71 (11.81)	128.61 (12.99)	126.61 (12.47)	123.05 (9.04)	*t*(97.44) = 1.73, *p* = 0.088
Nighttime, mm Hg	107.64 (13.77)	112.26 (8.61)	109.99 (11.59)	107.95 (11.29)	*t*(78.71) = 0.88, *p* = 0.381
Nocturnal dipping, mm Hg	15.18 (11.53)	14.56 (10.14)	14.87 (10.77)	15.11 (10.16)	*t*(80.78) = –0.11, *p* = 0.910
Diastolic blood pressure					
24-h, mm Hg	70.24 (9.37)	72.00 (8.21)	71.11 (8.80)	68.66 (4.81)	*t*(108.70) = 1.90, *p* = 0.060
Daytime, mm Hg	75.24 (8.68)	77.00 (8.87)	76.09 (8.76)	73.13 (6.18)	*t*(99.22) = 2.07, *p* = 0.041
Nighttime, mm Hg	59.00 (10.20)	61.09 (8.41)	60.07 (9.31)	59.74 (6.44)	*t*(99.18) = 0.22, *p* = 0.827
Nocturnal dipping, mm Hg	15.00 (9.37)	15.26 (7.80)	15.13 (8.53)	13.39 (8.10)	*t*(80.12) = 1.04, *p* = 0.302

BDI-II, Beck Depression Inventory-II; CBT, Cognitive behavioral therapy; CRP, C-reactive protein; HF-HRV, high-frequency heart rate variability; IL-6, interleukin 6; LF-HRV, low-frequency heart rate variability; MADRS, Montgomery–Asberg Depression Rating Scale; RMSSD, square root of the mean of the sum of the squares of differences between adjacent NN intervals; SDNN, a standard deviation of all NN intervals; TNF-*α*, tumor necrosis factor *α*; WL, waitlist.

Values are mean (s.d.) unless noted with percentage. Group differences were calculated using χ^2^ tests for categorical variables and *t* tests for continuous variables.

1 Winkler index for measuring individual socioeconomic status by combining information on education, income (monthly household net income), and occupation (ranging from 3 to 21).

2 Untransformed values are shown for ease of interpretation, although statistical comparisons were conducted on the square root transformed data.

### Main treatment effects

**Fig. 2.**
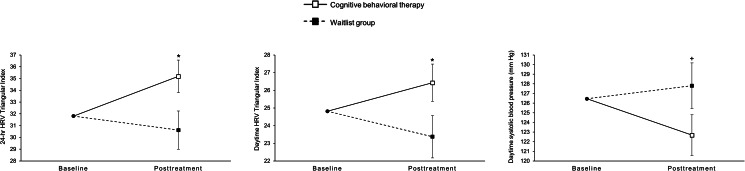
Treatment group differences in changes for cardiac measures from baseline to the end of treatment. *Note.* Values are estimated marginal means (standard errors) from constrained linear mixed models (see Table 2 for test statistics). HRV, heart rate variability. +*p* *<* 0.10 *p *<* 0.05.

Table 2 reports test statistics for differences in changes from baseline to the end of treatment between both treatment groups. Compared to patients in the WL group, patients who received CBT had stronger reductions in self-rated depressive symptoms (*d* = 0.52) and in clinician-rated depressive symptoms (*d* = 0.61). Changes in inflammatory markers and measures of heart rate did not significantly differ between patients in the CBT group and patients in the WL group. In terms of HRV measures which were reduced in depressed patients at baseline, the CBT group showed a significant increase in 24-h HRV triangular index (*d* = 0.48) and in daytime HRV triangular index (*d* = 0.43) from baseline to the end of treatment, compared to the WL group (shown in Fig. 2). Although 24-h LF-HRV was not significantly reduced in depressed patients at baseline, there was a trend increase in 24-h LF-HRV (*d* = 0.35) in the CBT group, compared to the WL group (see online Supplementary material Fig. S1). With respect to blood pressure measures which were increased (or marginally increased) in depressed patients at baseline, the CBT group showed a trend decrease in daytime SBP (*d* = 0.36), compared to patients in the WL group (shown in Fig. 2). There was no evidence for the main treatment effect in other variables of HRV or blood pressure. Compared to intention-to-treat analyses, analyses per protocol (results not shown) did not provide any substantial changes in the estimates and showed the same pattern of significance for treatment effects with one exception: the treatment effect for 24-h LF-HRV became significant in the per-protocol analysis (Intention-to-treat: *p* = 0.051; per-protocol: *p* = 0.0499).

**Table 2.** Associations between treatment groups and outcome measures over time. Results from constrained linear mixed models (Intention-to-treat analyses)

Outcomes	Baseline^a^	Change from baseline to the end of treatment				Treatment effect	
	Estimated mean (95% CI)	CBT group (*n* = 40)		WL group (*n* = 40)		Estimated differences in change (95% CI)	*p* value
		Estimated Change (95% CI)	*p* value	Estimated Change (95% CI)	*p* value		
Depression severity							
Self-rated, BDI-II	29.22 (27.37–31.08)	‒9.19 (‒12.27 to ‒6.12)	<0.001	‒3.38 (‒0.02 to 6.79)	0.051	‒5.81 (‒10.36 to ‒1.26)	0.013
Clinician-rated, MADRS	29.04 (27.59–30.49)	‒12.38 (‒16.04 to ‒8.71)	<0.001	‒‒5.16 (‒9.08 to ‒1.21)	0.011	‒7.22 (‒12.47 to ‒1.97)	0.008
Immunological markers							
SQRT CRP, *μ*g/ml	0.67 (0.59–0.76)	‒0.02 (‒0.13 to 0.09)	0.722	‒0.05 (‒0.17 to 0.07)	0.365	0.04 (‒0.12 to 0.19)	0.642
IL-6, pg/ml	0.52 (0.45–0.59)	0.11 (‒0.02 to 0.23)	0.084	004 (‒0.09 to 0.17)	0.561	0.07 (‒0.12 to 0.24)	0.427
TNF-*α*, pg/ml	1.07 (1.00–1.13)	0.01 (‒0.08 to 0.08)	0.907	‒0.07 (‒0.16 to 0.02)	0.112	0.08 (‒0.04 to 0.19)	0.193
Heart rate							
24-h, beats/min	77.78 (75.57–79.98)	‒3.31 (‒5.94 to ‒0.68)	0.015	‒0.83 (‒3.89 to 2.23)	0.589	‒2.49 (‒6.34 to 1.37)	0.200
Daytime, beats/min	85.67 (83.33–88.02)	‒3.24 (‒6.18 to ‒0.30)	0.032	‒2.12 (‒5.48 to 1.25)	0.213	‒1.12 (‒5.47 to 3.23)	0.606
Nighttime, beats/min	64.31 (62.1–69.83)	0.10 (‒3.15 to 3.34)	0.953	1.46 (‒2.38 to 5.30)	0.447	‒1.37 (‒6.31 to 3.58)	0.580
Heart rate variability							
Triangular index							
24-h	31.81 (29.91–33.72)	3.37 (0.46–6.28)	0.024	‒1.19 (‒4.58 to 2.20)	0.483	4.56 (0.40–8.72)	0.033^b^
Daytime	24.81 (23.26–26.36)	1.62 (‒0.54 to 3.77)	0.139	‒1.44 (‒3.87 to 0.99)	0.239	3.06 (0.02–6.10)	0.049^b^
Nighttime	20.03 (18.38–21.68)	1.85 (‒0.66 to 4.35)	0.145	‒1.03 (‒3.96 to 1.90)	0.484	2.88 (‒0.80 to 6.55)	0.122
HF-HRV							
24-h, ms^2^	812.00 (662.62–961.38)	58.96 (‒76.63 to 194.54)	0.386	‒64.80 (‒224.28 to 94.69)	0.418	123.75 (‒79.37 to 326.88)	0.226
Daytime, ms^2^	452.77 (372.78–532.76)	141.84 (41.43–242.26)	0.007	58.46 (‒56.03 to 172.96)	0.308	83.381 (‒67.60 to 234.37)	0.271
Nighttime, ms^2^	1300.74 (1033.52–1567.96)	‒73.97 (‒385.64 to 237.70)	0.635	‒122.02 (‒487.08 to 243.04)	0.504	48.05 (‒411.16 to 507.26)	0.834
LF-HRV							
24-h, ms^2^	1463.10 (1284.57–1641.63)	107.87 (‒55.90 to 271.64)	0.191	‒137.85 (‒330.92 to 55.22)	0.157	245.72 (‒1.42 to 492.86)	0.051
Daytime, ms^2^	1200.61 (964.14–1337.09)	120.77 (‒12.95 to 254.49)	0.075	28.21 (‒126.15 to 182.56)	0.731	92.57 (‒110.33 to 295.46)	0.361
Nighttime, ms^2^	1726.37 (1481.83–1970.90)	183.54 (‒205.97 to 503.04)	0.348	‒63.162 (‒523.32 to 397.00)	0.783	246.70 (‒343.44 to 836.84)	0.404
RMSSD							
24-h, ms	40.17 (36.28–44.07)	2.22 (‒1.45 to 5.89)	0.229	‒0.05 (‒4.38 to 4.29)	0.982	2.27 (‒3.32 to 7.85)	0.417
Daytime, ms	30.59 (27.63–33.55)	3.38 (0.30–6.46)	0.032	1.76 (‒1.71 to 5.22)	0.313	1.63 (‒2.84 to 6.09)	0.467
Nighttime, ms	54.75 (48.10–61.41)	‒0.68 (‒7.91 to 6.55)	0.851	‒1.52 (‒9.98 to 6.94)	0.719	0.84 (‒9.77 to 11.46)	0.873
SDNN							
24-h, ms	158.60 (149.72–167.49)	‒0.11 (‒10.12 to 9.91)	0.983	‒6.86 (‒18.62 to 4.90)	0.246	6.75 (‒8.14 to 21.64)	0.365
Daytime, ms	116.72 (109.27–124.16)	6.51 (‒4.55 to 17.57)	0.243	‒3.97 (‒16.47 to 8.53)	0.527	10.48 (‒5.31 to 26.27)	0.188
Nighttime, ms	111.92 (103.36–120.49)	8.27 (‒4.84 to 21.43)	0.210	‒0.17 (‒15.71 to 15.38)	0.983	8.46 (‒11.58 to 28.51)	0.400
Blood pressure							
Systolic blood pressure							
24-h, mm Hg	121.04 (118.17–123.91)	‒2.00 (‒5.13 to 1.12)	0.204	0.92 (‒2.66 to 4.50)	0.608	‒2.92 (‒7.59 to 1.74)	0.213
Daytime, mm Hg	126.46 (123.59–129.32)	‒3.78 (‒7.67 to 0.12)	0.057	1.35 (‒3.14 to 5.84)	0.549	‒5.13 (‒10.92 to 0.66)	0.081
Nighttime, mm Hg	109.83 (107.04–112.62)	‒0.87 (‒4.76 to 3.02)	0.655	2.68 (‒1.82 to 7.17)	0.237	‒3.55 (‒9.31 to 2.22)	0.222
Nocturnal dipping^c^, mm Hg	14.79 (12.20–17.38)	‒0.76 (‒4.90 to 3.38)	0.594	1.16 (‒3.50 to 5.83)	0.620	‒1.92 (‒7.61 to 3.76)	0.500
Diastolic blood pressure							
24-h, mm Hg	70.91 (68.87–72.94)	‒1.58 (‒3.96 to 0.79)	0.187	0.02 (‒2.67 to 2.71)	0.986	‒1.61 (‒5.00 to 1.79)	0.347
Daytime, mm Hg	76.10 (74.08–78.11)	‒2.89 (‒5.75 to ‒0.03)	0.048	0.18 (‒3.12 to 3.48)	0.914	‒3.07 (‒7.21 to 1.08)	0.143
Nighttime, mm Hg	59.95 (57.72–62.18)	0.27 (‒2.83 to 3.36)	0.864	0.87 (‒2.59 to 4.33)	0.617	‒0.61 (‒4.80 to 3.59)	0.773
Nocturnal dipping^c^, mm Hg	15.20 (13.04–17.15)	‒2.88 (‒6.29 to 0.53)	0.097	0.97 (‒2.91 to 4.85)	0.618	‒3.85 (‒8.66 to 0.96)	0.114

BDI-II, Beck Depression Inventory-II; CBT, Cognitive behavioral therapy; CRP, C-reactive protein; HF-HRV, high-frequency heart rate variability; IL-6, interleukin 6; LF-HRV, low-frequency heart rate variability; MADRS, Montgomery–Asberg Depression Rating Scale; RMSSD, square root of the mean of the sum of the squares of differences between adjacent NN intervals; SDNN, standard deviation of all NN intervals; SQRT, square root transformed; TNF-*α*, tumor necrosis factor *α*; WL, waitlist.

a Baseline-adjusted results; all patients are contained in one group at baseline.

b A finding is not observed after adjustment with a false discovery rate of 10% (see methods section for details on false discovery adjustment).

c Models include corresponding 24-h blood pressure as a time-varying covariate.

### Moderated treatment effects

**Fig. 3.**
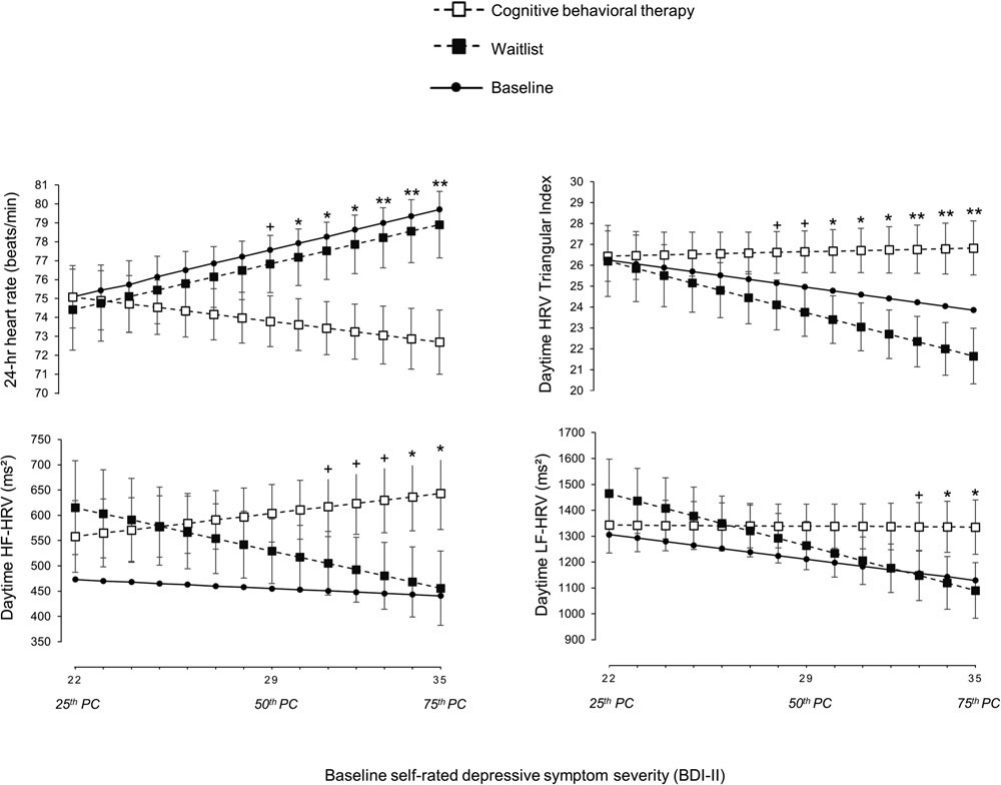
Baseline self-rated depressive symptom severity (i.e. BDI-II) as moderator of differences in changes in cardiac measures from baseline to the end of treatment. *Note.* Estimated marginal means (standard errors) from constrained linear mixed models are plotted from lower (25^th^ percentile) to higher (75^th^ percentile) levels of the moderator (see Table 3 for test statistics). HRV, heart rate variability; HF-HRV, high-frequency heart rate variability; LF-HRV, low-frequency heart rate variability. +*p* *<* 0.10 *p *<* 0.05 **p *<* 0.01.

Table 3 shows results for moderation analyses with baseline self-rated (i.e. BDI-II) and clinician-rated (i.e. MADRS) depressive symptom severity as a predictor for changes in biological measures from baseline to the end of treatment. In cases where there were any significant or trend moderating effects (*p* < 0.10), differences between changes were plotted from lower (25^th^ percentile) to higher (75^th^ percentile) levels of the moderator to explore the nature of the moderation. Figure 3 plots moderating effects for biological measures which were altered in patients with MD at baseline (see online Supplementary material Fig. S2 for all other moderating effects).

**Table 3.** Depressive symptom severity as moderator for associations between treatment groups and outcome measures over time. Results from constrained linear mixed models (Intention-to-treat analyses)

	Moderator			
Outcomes	Self-rated, BDI-II		Clinician rated, MADRS	
	Estimate (95% CI)	*p* value	Estimate (95% CI)	*p* value
Immunological markers				
SQRT CRP, *μ*g/ml	0.01 (‒0.004 to 0.03)	0.124	0.02 (‒0.07 to 0.04)	0.175
IL-6, pg/ml	‒0.02 (‒0.04 to 0.01)	0.147	0.01 (‒0.02 to 0.03)	0.645
TNF-*α*, pg/ml	‒0.002 (‒0.02 to 0.01)	0.782	‒0.001 (‒0.02 to 0.02)	0.900
Heart rate				
24-h, beats/min	‒0.53 (‒0.95 to ‒0.11)	0.014^a^	‒0.05 (‒0.60, to 0.51)	0.862
Daytime, beats/min	‒0.41 (‒0.94 to 0.11)	0.120	0.36 (‒0.28 to 1.00)	0.261
Nighttime, beats/min	‒0.52 (‒1.10 to 0.07)	0.081	‒0.03 (‒0.77 to 0.71)	0.927
Heart rate variability				
Triangular index				
24-h	0.49 (‒0.001 to 0.98)	0.050	‒0.27 (‒0.89 to 0.35)	0.389
Daytime	0.38 (0.01–0.75)	0.043^a^	‒0.34 (‒0.79 to 0.10)	0.126
Nighttime	0.26 (‒0.20 to 0.71)	0.260	‒0.15 (‒0.70 to 0.39)	0.576
HF-HRV				
24-h, ms^2^	19.85 (‒3.00 to 41.99)	0.078	10.70 (‒12.65 to 34.06)	0.360
Daytime, ms^2^	18.79 (0.66–36.93)	0.043^a^	3.02 (‒17.93 to 23.97)	0.772
Nighttime, ms^2^	35.65 (‒16.27 to 87.57)	0.173	8.82 (‒50.91 to 68.54)	0.767
LF-HRV				
24-h, ms^2^	17.24 (‒10.41 to 44.89)	0.215	5.74 (‒26.94 to 38.42)	0.724
Daytime, ms^2^	28.24 (4.20–52.27)	0.023^a^	13.78 (‒12.75 to 40.31)	0.299
Nighttime, ms^2^	45.39 (‒22.03 to 112.81)	0.181	5.82 (‒79.57 to 91.21)	0.891
RMSSD				
24-h, ms	0.52 (‒0.03 to 1.08)	0.065	0.41 (‒0.28 to 1.10)	0.235
Daytime, ms	0.46 (‒0.07 to 0.99)	0.086	‒0.04 (‒0.67 to 0.59)	0.908
Nighttime, ms	0.87 (‒0.28 to 2.02)	0.136	0.51 (‒0.96 to 1.98)	0.485
SDNN				
24-h, ms	1.98 (0.23–3.67)	0.023^a^	‒0.23 (‒2.46 to 2.01)	0.840
Daytime, ms	1.81 (‒0.11 to 3.73)	0.065	‒1.96 (‒4.31 to 0.39)	0.100
Nighttime, ms	3.00 (0.69–5.31)	0.012^a^	0.16 (‒2.88 to 3.21)	0.916
Blood pressure				
Systolic blood pressure				
24-h, mm Hg	‒0.14 (‒0.74 to 0.47)	0.646	‒0.08 (‒0.83 to 0.66)	0.822
Daytime, mm Hg	‒0.11 (‒0.87 to 0.65)	0.771	‒0.30 (‒1.23 to 0.62)	0.513
Nighttime, mm Hg	0.004 (‒0.68 to 0.69)	0.991	0.03 (‒0.86 to 0.92)	0.949
Nocturnal dipping^b^, mm Hg	‒0.23 (‒0.89 to 0.44)	0.496	‒0.44 (‒1.29 to 0.41)	0.303
Diastolic blood pressure				
24-h, mm Hg	‒0.13 (‒0.57 to 0.31)	0.555	‒0.01 (‒0.55 to 0.54)	0.978
Daytime, mm Hg	‒0.04 (‒0.58 to 0.50)	0.880	‒0.06 (‒0.72 to 0.60)	0.866
Nighttime, mm Hg	‒0.07 (‒0.59 to 0.45)	0.787	0.28 (‒0.36 to 0.92)	0.381
Nocturnal dipping^b^, mm Hg	0.04 (‒0.53 to 0.62)	0.882	‒0.36 (‒1.06 to 0.35)	0.315

BDI-II, Beck Depression Inventory-II; CBT, Cognitive behavioral therapy; CRP, C-reactive protein; HF-HRV, high-frequency heart rate variability; IL-6, interleukin 6; LF-HRV, low-frequency heart rate variability; MADRS, Montgomery–Asberg Depression Rating Scale; RMSSD, square root of the mean of the sum of the squares of differences between adjacent NN intervals; SDNN, a standard deviation of all NN intervals; SQRT, square root transformed; TNF-*α*, tumor necrosis factor *α*; WL, waitlist.

a A finding is observed after adjustment with a false discovery rate of 10% (see methods section for details on false discovery adjustment).

b Models include corresponding 24-h blood pressure as a time-varying covariate.

Self-rated (but not clinician-rated) baseline depressive symptom severity significantly moderated differences between changes in 24-h heart rate. In patients with more severe depressive symptoms at study entry, there was a stronger decrease in 24-h heart rate from baseline to the end of treatment in the CBT group, compared to the WL group (see Fig. 3). A significant treatment effect on 24-h heart rate in favor of CBT over WL emerged with a baseline severity score of BDI-II = 30 (*d* = 0.39). At the 75^th^ percentile (BDI = 35), the effect on 24-h heart rate was moderate (*d* = 0.57). Although not altered at study entry, there was also a trend for this moderating effect in terms of changes in nighttime heart rate (see online Supplementary material Fig. S2).

In terms of HRV measures which were impaired (or marginally impaired) in depressed patients at baseline, higher self-rated depressive symptom severity at baseline was associated with significant improvements in daytime HRV triangular index, daytime HF-HRV, and daytime LF-HRV from baseline to the end of treatment in the CBT compared to the WL group (see Fig. 3). A significant treatment effect in favor of CBT over WL emerged with a baseline severity score of BDI-II = 30 for daytime HRV triangular index (*d* = 0.48) and of BDI-II = 34 for daytime HF-HRV (*d* = 0.39) and daytime LF-HRV (*d* = 0.34). At the 75^th^ percentile (BDI = 35), treatment effects were estimated to be moderate for daytime HRV triangular index (*d* = 0.62) and small for daytime HF-HRV (*d* = 0.41) and daytime LF-HRV (*d* = 0.37). There was also a trend for this moderating effect in 24-h HRV triangular index, daytime RMSSD and daytime SDNN (see online Supplementary material Fig. S2). Although not altered in depressed patients at baseline, similar moderating effects were observed for 24-h SDNN and nighttime SDNN. A significant treatment effect in favor of CBT over WL emerged with a baseline severity score of BDI-II = 34 for 24-h SDNN (*d* = 0.40) and nighttime SDNN (*d* = 0.43). At the 75^th^ percentile (BDI = 35), treatment effects were estimated to be small for 24-h SDNN (*d* = 0.43) and nighttime SDNN (*d* = 0.46) (see online Supplementary material Fig. S2). Depressive symptom severity did not moderate the impact of treatment on inflammatory markers. Compared to intention-to-treat analyses, analyses per protocol (results not shown) did not provide any substantial changes in the estimates and showed the same pattern of significance for moderated treatment effects with one exception: the moderated treatment effect (i.e. self-rated depressive symptom severity) for 24-h HRV triangular index became significant in the per-protocol analysis (Intention-to-treat: *p* = 0.050; per-protocol: *p* = 0.049).

### Correlations between changes in depressive symptom severity and biological outcomes

In cases where cLDA resulted in any significant or trend effects (*p* < 0.10), we performed post-hoc intention-to-treat correlational analyses with maximum likelihood estimation to examine whether a reduction in depressive symptom severity correlated with corresponding improvements in biological outcomes in the CBT group. A decline in self-rated depressive symptom severity from baseline to the end of CBT significantly correlated with a decrease in 24-h heart rate (*r* = 0.35; *p* = 0.010) and tended to correlate with a decrease in nighttime heart rate (*r* = 0.25; *p* = 0.076). In addition, decrease in self-rated depressive symptom severity significantly correlated with improvement in 24-h HF-HRV (*r* = −0.43; *p* < 0.001), daytime HF-HRV (*r* = −0.35; *p* = 0.004), 24-h RMSSD (*r* = −0.49; *p* < 0.001) and daytime RMSSD (*r* = −0.41; *p* = 0.001) but not with changes in measures of SDNN, LF-HRV and HRV triangular index, as well as daytime SBP (all *p*s ⩾ 0.1). Correlations between changes in clinician-rated depressive symptom severity and changes in cardiac measures were non-significant (all *p*s ⩾ 0.1).

### A posterori multiplicity adjustments

As outlined in the methods section, all results are not adjusted for multiple testing and should be considered exploratory (Althouse, 2016; Bender & Lange, 2001; Rothman, 1990; Rubin, 2017). However, because there may not always be consensus on the need for multiplicity adjustment in exploratory studies, we decided to provide separate post-hoc multiplicity adjustments with a false discovery rate (FDR) of 10% in cases where at least one significant treatment effect was observed within a reasonable family of potentially interrelated HR and HRV outcomes. More specifically, we separately adjusted within the domain of 24-h HR and HRV measures, within the domain of daytime HR and HRV measures, as well as within the domain of nighttime HR and HRV measures via the less-conservative two-stage step-up method of Benjamini, Krieger, and Yekutieli (2006) using Prism 9 (GraphPad) for Windows. As shown in Table 2, the main treatments effects on 24-h and daytime HRV triangular index could not be observed after FDR adjustments. As seen in Table 3, all originally significant moderated treatment effects would also be prominent after FDR correction. Importantly, multiplicity adjustments were conducted a posteriori and thus the sample size in this study may not account for these adjustments.

## Discussion

This exploratory RCT examined whether CBT is accompanied by reductions in cardiac risk biomarkers in antidepressant-free patients with MD. Biological outcomes included a wide range of measures with potential predictive value for CVD such as heart rate (Kannel et al., 1987), indices of HRV (Hillebrand et al., 2013), blood pressure (Lewington et al., 2002), as well as inflammatory markers (i.e. CRP, IL-6, and TNF-*α*) (Avan et al., 2018; Pearson, 2003; Subirana et al., 2018). Compared to WL, CBT was associated with a significant increase in 24-h and daytime HRV triangular index and a borderline significant increase in 24-h LF-HRV, as well as a trend decrease in daytime SBP. Self-rated depressive symptom severity at study entry moderated (or tended to moderate) improvements through CBT for 24-h and daytime heart rate, and for several indices of HRV, particularly those daytime measures which were altered in depressed patients at study entry. With increasing symptom severity, CBT increased or tended to increase these indices of HRV and reduced heart rate, compared to the control condition. Inflammatory effects were not observed in this trial.

At study entry, the 24-h and daytime HRV triangular index, a robust geometric measure of overall HRV (Stapelberg, Neumann, Shum, McConnell, & Hamilton-Craig, 2018; Vila, Lado, & Cuesta-Morales, 2019; Ziegler et al., 1999), was reduced in patients with MD. Compared to the control condition, CBT increased the 24-h and daytime HRV triangular index. Several studies suggest that overall HRV predict cardiovascular health outcomes in populations without known CVD at study entry, as well as in clinical populations (Fang, Wu, & Tsai, 2020a, 2020b; Hämmerle et al., 2020; Hillebrand et al., 2013). Although our finding needs confirmatory replication, an improvement of overall HRV during CBT may suggest that this common form of psychotherapy does not only improve depressive symptom severity but also cardiovascular health. Given the increased incidence of CVD in patients with MD, it is of clinical relevance whether common treatments affect cardiovascular health (Gan et al., 2014; Harshfield et al., 2020; Li et al., 2019; Wu & Kling, 2016). Our finding may thus inspire future research to include the HRV triangular index when evaluating biological effects of psychotherapy in MD, and also to examine whether potential improvements in overall HRV during treatment translate into reduced risk for CVD.

Although CBT numerically increased all other indices of HRV, differences in changes between CBT and WL were not significant. One explanation for this finding might be, that the triangular index is more reliable than non-geometric measures of HRV in terms of high intraindividual reproducibility and thus more appropriate for prospective research (Stapelberg et al., 2018; Vila et al., 2019; Ziegler et al., 1999). The major disadvantage of the triangular index is that time-consuming long-term recordings should be used to ensure valid calculation (Heart Rate Variability: Standards of Measurement, Physiological Interpretation and Clinical Use. Task Force of the European Society of Cardiology and the North American Society of Pacing and Electrophysiology, 1996). However, because of its robustness and responsiveness in this trial, the triangular index might be a good measure to evaluate changes in overall HRV through psychological treatment.

The observation that self-rated depressive symptom severity, assessed by the BDI, moderated treatment effects on heart rate and daytime HRV is in line with findings from an earlier pre-post study in depressed patients with coronary heart disease (Carney et al., 2000). In this previous work, patients were classified according to their BDI score as mildly depressed or moderately to severely depressed. The main result was that heart rate and daytime RMSSD improved significantly in the moderately to severely depressed patients but remained unchanged in the mildly depressed patients (Carney et al., 2000). This earlier study together with our present RCT suggests that potential changes in heart rate and especially in daytime measures of HRV are primarily observable in patients with more severe depressive symptoms. Patients with more severe depressive symptoms thus seem to improve their cardiovascular risk profile the most. This finding is of particular interest since also the risk for CVD increases with increasing self-rated depressive symptom severity (Harshfield et al., 2020) and an improvement of HRV during treatment might thus be of stronger prognostic relevance in more severe depression. Higher depression scores have greater potential for change than lower scores. If the degree of change in depressive symptoms relates to changes in heart rate and HRV, one would expect that a reduction in depressive symptom severity relates to improvements in heart rate and HRV during treatment. Indeed, the present study found that a reduction in self-rated depressive symptom severity through CBT significantly correlated with improvements in HF-HRV, RMSSD and heart rate. Importantly, clinician-rated depressive symptoms, as assessed by the MADRS, did neither moderate biological treatment effects nor correlate with changes in a cardiac measure in CBT. One explanation for these diverging findings may be that the BDI, in contrast to the MADRS, has been conceptualized to reflect outcomes of psychotherapy (Demyttenaere & De Fruyt, 2003) and may thus stronger relate to the biological effects of CBT. Moreover, expert ratings are prone to over-estimate clinical improvements (Cuijpers, Li, Hofmann, & Andersson, 2010; Rief et al., 2009). For future psychotherapeutic trials, we suggest considering moderating effects of patients’ self-rated symptom severity when studying changes in heart rate and HRV.

In the present study, patients exhibited increased levels of inflammatory levels at study entry. There was no evidence for reductions in these markers through CBT. A previous RCT with partially medicated patients with depression and elevated cardiovascular risk did also not observe an effect of CBT on CRP (Taylor et al., 2009). Another RCT with partially medicated patients found that CBT reduced CRP (but not IL-6) only in patients with increased baseline CRP levels, and only in combination with physical exercise (Euteneuer et al., 2017). On the other hand, reductions in IL-6 and TNF-*α* during CBT (but not during Narrative Cognitive Therapy) have been observed in a study with young patients with MD (18 to 29 years old), but this RCT did only analyze within-group changes and provided no intention-to-treat analyses for differences in changes between both treatment groups (Moreira et al., 2015). Based on all these findings, there may be little or no evidence for inflammatory effects of standard CBT in the context of depression, although some findings in other populations suggest that CBT may improve inflammation, for example, in insomnia (Irwin et al., 2015) or fibromyalgia (Zabihiyeganeh et al., 2019). Even if inflammation may neither be necessary nor sufficient to induce or sustain depression in general (Kiecolt-Glaser et al., 2015), a potential insufficiency of CBT to improve low-grade inflammation strengthens the importance of research efforts to develop more specific anti-inflammatory treatments for subgroups of patients (Jones et al., 2020).

Although findings for associations between depression and blood pressure alterations are mixed (Gould & Beaudreau, 2013; Hildrum et al., 2007; Rogeness, Cepeda, Macedo, Fisher, & Harris, 1990), depression has been shown to predict the development of hypertension (Ginty et al., 2013; Meng et al., 2012). In addition, research using long-term assessment of blood pressure (i.e. 7 days) suggests increased SBP and DBP, as well as reduced nocturnal blood pressure dipping in depressed individuals (Shinagawa et al., 2002). Compared to non-clinical controls, patients in the present study showed significantly increased 24-h SBP and daytime DBP, as well as marginally higher daytime SBP and 24-h DBP. Although these alterations numerically improved in the CBT group compared to the WL group, there was only a trend effect for daytime SBP. To the best of our knowledge, there are no previous RCTs that evaluate CBT effects on ambulatory blood pressure in patients with MD. So, this finding is of potential interest but needs further investigation.

Notwithstanding the strengths of the present study, such as the inclusion of a non-clinical control group, the randomized controlled design, the comprehensive assessment of potential cardiac risk biomarkers, the inclusion of patients without antidepressant medication, and the use of 24-h long-term recordings for cardiac measures instead of short-term recordings, limitations need to be reflected. First, given the exploratory nature of this trial, further research is necessary to confirm the observed findings. In particular, treatment effects for the HRV triangular index may have occurred by chance. Second, our sample consists of outpatients with MD who were eligible for psychological treatment. Thus, findings may not generalize to other samples of depressed patients (e.g. MD patients with psychotic features). Third, we did not assess patients` experience with CBT, a factor which might of relevance for treatment responsiveness. Fourth, although studies on depression and dysregulation of the hypothalamic–pituitary–adrenal (HPA) stress axis are less consistent (Dedovic & Ngiam, 2015; Psarraki et al., 2021; Stetler & Miller, 2011), there is meanwhile evidence for a prospective association between higher levels of morning plasma cortisol and incident CVD (Crawford et al., 2019). It might thus have been useful to include HPA-axis biomarkers in this study. Moreover, this RCT was not designed to examine specific mechanisms (as described in the introduction section) and the relative contribution of specific components of CBT to potential biological effects, which may be of interest for future dismantling studies. Finally, despite longitudinal evidence linking heart rate and HRV to cardiovascular risk, the clinical implications of the present study are not clear. The most important question is whether improving biological cardiac risk factors in patients with MD will translate into reduced risk for CVD. Given the lack of studies which focus on such long-term effects of psychological treatment, it is not possible to answer this question.

To conclude, this study found that CBT for MD is accompanied by an increase in overall HRV, as indexed by the triangular index. If this exploratory result is replicated in confirmatory studies, this could imply that the cardiovascular risk profile is reduced after successful CBT. Moreover, self-rated depressive symptom severity was identified as a potential moderator for improvements in several indices of heart rate and HRV. The findings provide new insights into biological effects of psychological treatment against depression.

## Supplementary material

For supplementary material accompanying this paper visit https://doi.org/10.1017/S0033291722000447. click here to view supplementary material
