# Supplementary material for: Does psychological treatment of major depression reduce cardiac risk biomarkers? An exploratory randomized controlled trial
Source: Psychol Med. 2022 Mar 2;53(8):3735–49. doi: 10.1017/S0033291722000447 (PMC10277774; doi:10.1017/S0033291722000447)
Supplement: Supplementary file 1 [file S0033291722000447sup.zip › S0033291722000447sup001.docx]

Supplementary material

**Does Psychological Treatment of Major Depression Reduce Cardiac Risk Biomarkers? An Exploratory Randomized Controlled Trial**

Euteneuer et al.

**Recruiting, Setting and Intervention**

**Figure S1**

**Figure S2**

**CONSORT 2010 checklist**

**Recruiting, Setting and Intervention**

Patients were recruited via the Outpatient Clinic for Psychological Interventions at the University of Marburg, via advertisements and leaflets in pharmacies and doctors' waiting rooms. Non-clinical controls were recruited via advertisements, university email lists, and press releases in local newspapers. After prescreening via phone, all patients underwent an initial session that included structural clinical interviewing, socio-demographic and psychological assessment, and approving inclusion/exclusion criteria. All participants gave oral and written informed consent.

Inclusion criteria for patients were a DSM-IV diagnosis of MD, a Beck Depression Inventory (BDI)-II score of ≥ 14 (indicating clinically relevant depressive symptoms), and the ability to fluently read and answer German self-rating scales and questionnaires. Exclusion criteria were the use of psychotropic medication (including antidepressants) during the last 14 days or cardiac drugs, a diagnosis of cardiovascular disease, chronic diseases (e.g., arthritis, asthma, cancer, chronic obstructive pulmonary disease, diabetes, neurological illness), psychotic symptoms, injuries and infections during the last 14 days, alcohol and/or drug abuse, current pregnancy and lactation in women, and any mental disorders according to DSM-IV in non-clinical controls.

To detect a small effect of group x time from baseline to posttreatment, with a statistical power of 1−β= 0.85 and a level of significance of α<.05, a sample size of at least 60 needed to be included when assuming that correlations between repeated measures are moderate. A sample size of *n* = 80 was preregistered and utilized to compensate for an estimated 25% dropout rate (Hans & Hiller, 2013).

Patients participated in 50 minutes of individual manualized psychotherapy weekly for 14 weeks. The treatment took place in the Outpatient Clinic for Psychological Interventions of the University of Marburg, Germany. A high number of therapists conducting a treatment may help to improve the generalizability of trial results. Therefore, we recruited a total of 29 psychologists to participate in this trial. All therapists were clinical psychologists (M.Sc. degree) with advanced or completed postgraduate clinical training in psychotherapy (which is a 3-year postgraduate training in psychotherapy after the M.Sc. degree). The CBT treatment was based on a common CBT manual and structured through phases typically used in CBT(Hautzinger, 2003). During an initial phase (Weeks 1–4), patients received psychoeducation on MD and the relationship between thoughts, feelings, and behavior. Additional elements were case conceptualization (i.e., assessment of individual risk factors for depression) and development of treatment goals. During a second phase (Weeks 5–8), patients received behavioral activation followed by cognitive therapy, individual skills training (i.e., problem solving, relaxation, social skills), and relapse prevention (Weeks 9–14). Patients in the WL group did not receive any treatment during the 14 weeks and were transferred to standard psychotherapy afterwards. According to the original protocol, this study had aimed at collecting data at 2 months follow-up after the intervention/waiting time. However, the 2 month follow-up was cancelled during the study phase due to feasibility problems within a natural treatment environment.

**References**

Hans, E., & Hiller, W. (2013). Effectiveness of and dropout from outpatient cognitive behavioral therapy for adult unipolar depression: a meta-analysis of nonrandomized effectiveness studies. *Journal of Consulting and Clinical Psychology*, *81*(1), 75–88. https://doi.org/10.1037/a0031080

Hautzinger, M. (2003). Kognitive Verhaltenstherapie bei Depressionen [Cognitive behavioral therapy for depression]. *Weinheim: Psychologie Verlags Union*.

**Figure S1**

**
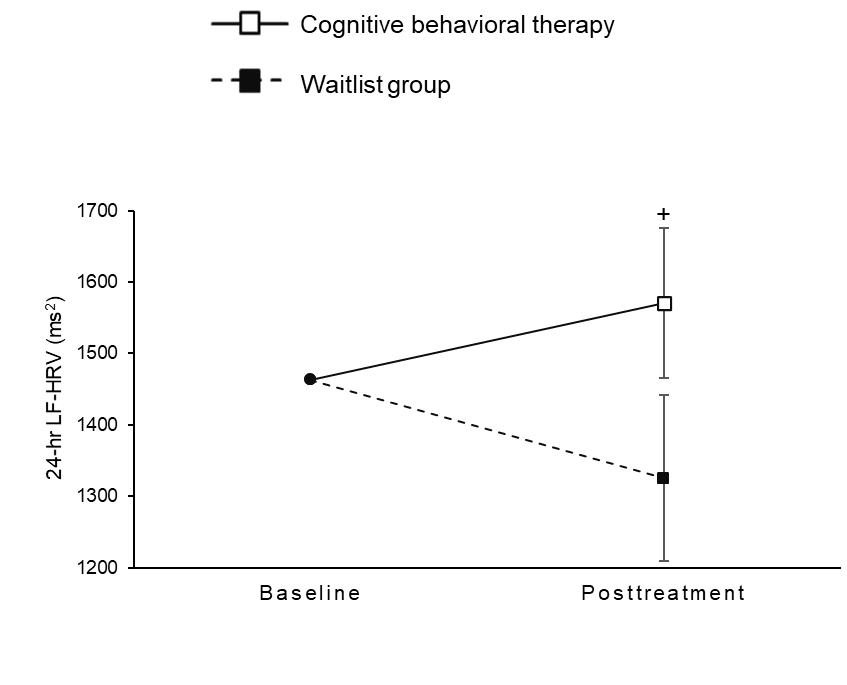
***Treatment group differences in changes for low-frequency heart rate variability (LF-HRV) from baseline to the end of treatment*

*Note.* Values are estimated marginal means (standard errors) from constrained linear mixed models (see Table 2 for test statistics). +*p <* .10.

**Figure S2**

*Baseline self-rated depressive symptom severity (i.e., BDI-II) as moderator of differences in changes in cardiac measures from baseline to the end of treatment*


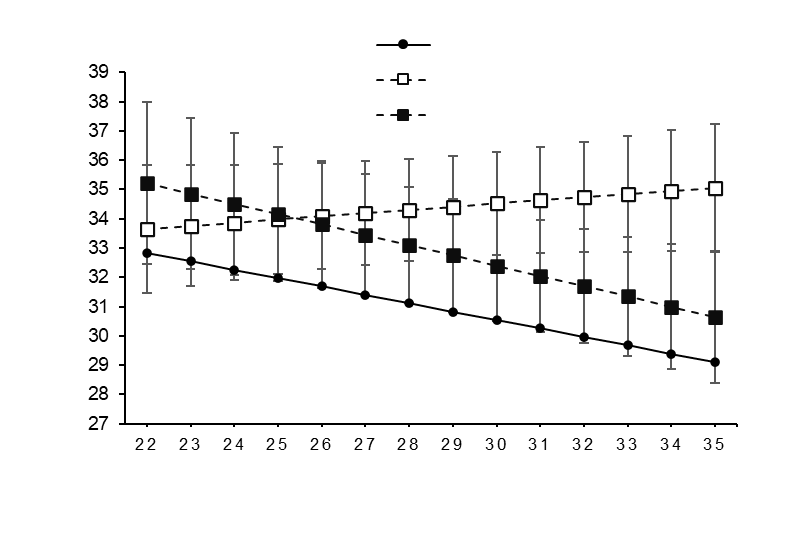

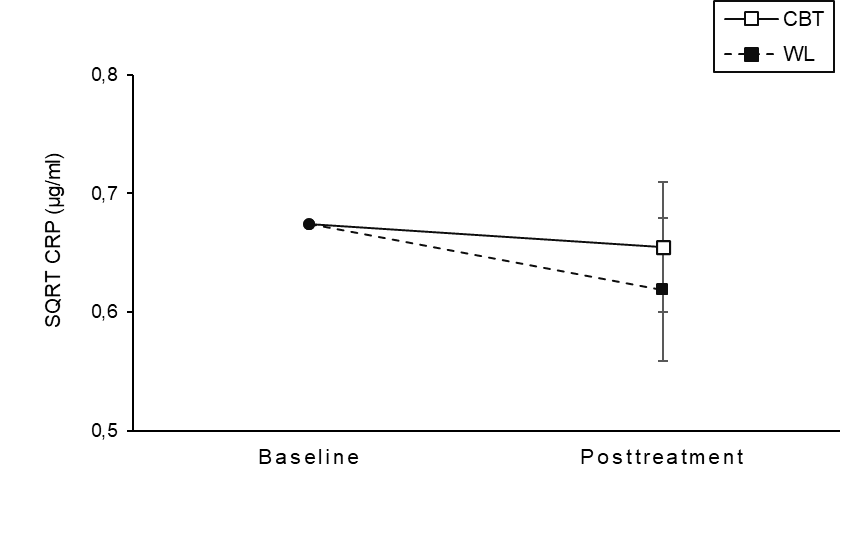


Cognitive behavioral therapy

Waitlist

Baseline


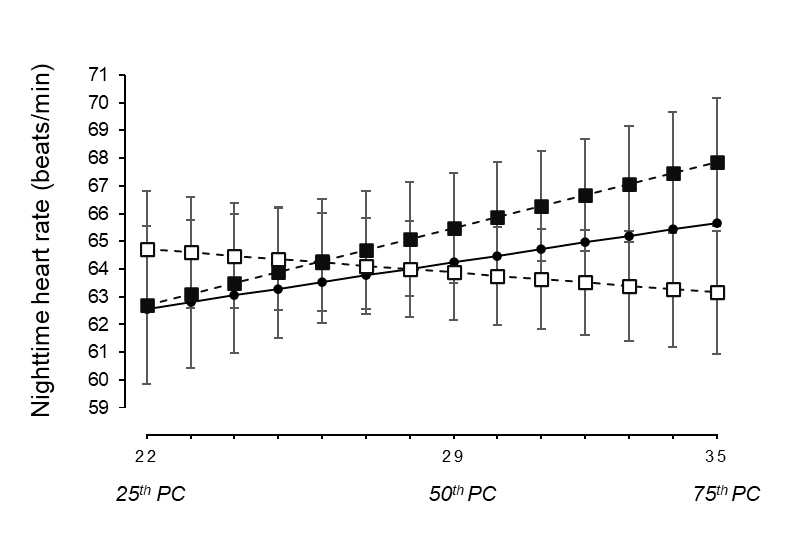

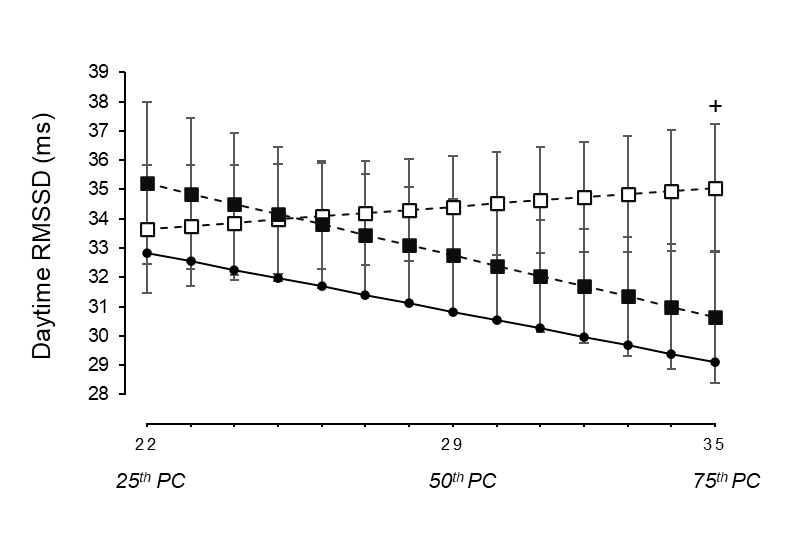

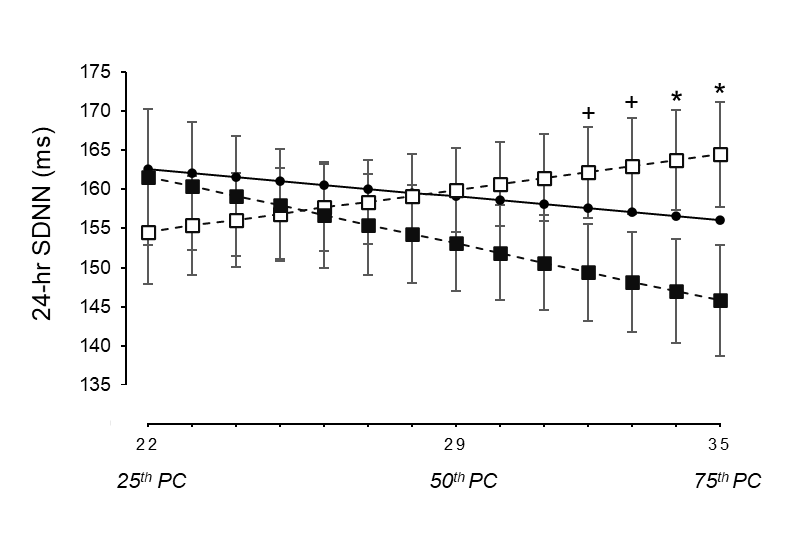

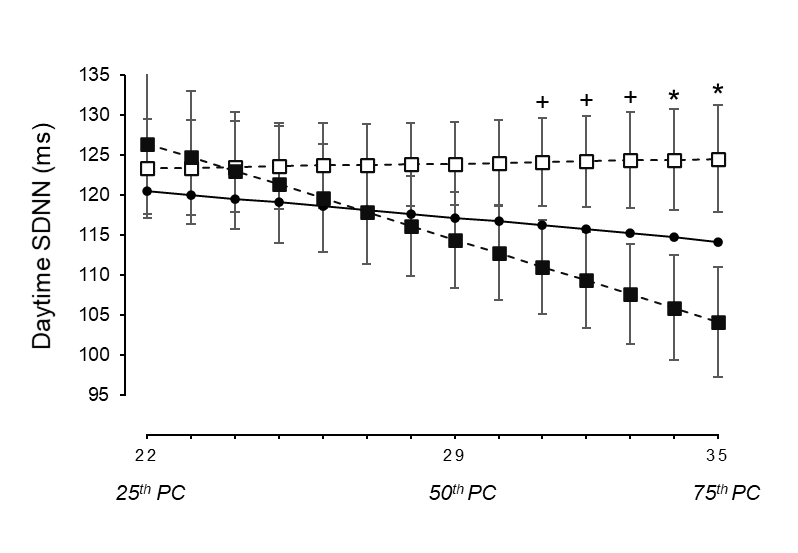

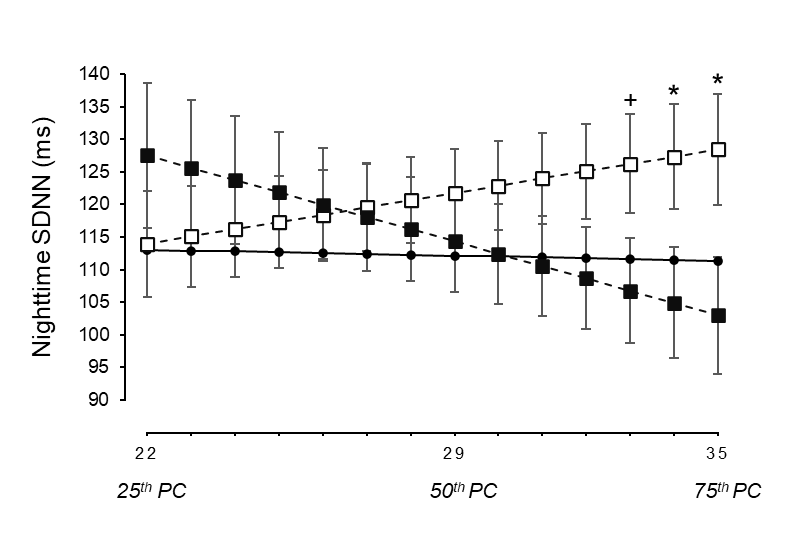

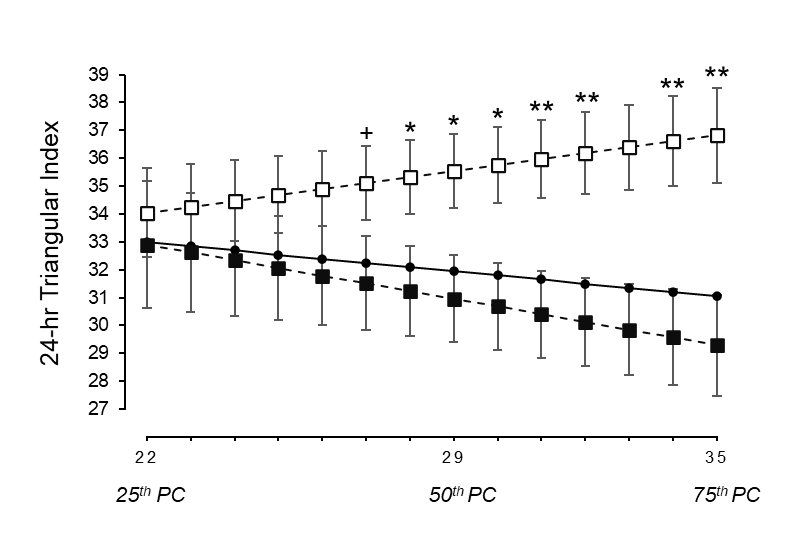


Baseline self-rated depressive symptom severity (BDI-II)

*Note.* Estimated marginal means (standard errors) from constrained linear mixed models are plotted from lower (25^th^ percentile) to higher (75^th^ percentile) levels of the moderator (see Table 3 for test statistics). HRV, heart rate variability; RMSSD, square root of the mean of the sum of the squares of differences between adjacent NN intervals; SDNN, standard deviation of all NN intervals. + *p <* .10 **p <* .05 ***p <* .01.


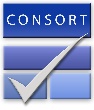
CONSORT 2010 checklist of information to include when reporting a randomised trial*

| Section/Topic | Item No | Checklist item | Reported on page No |
| --- | --- | --- | --- |
| Title and abstract | | | |
|  | 1a | Identification as a randomised trial in the title | 1 |
|  | 1b | Structured summary of trial design, methods, results, and conclusions (for specific guidance see CONSORT for abstracts) | 2 |
| Introduction | | | |
| Background and objectives | 2a | Scientific background and explanation of rationale | 3-5 |
|  | 2b | Specific objectives or hypotheses | 3-5 |
| Methods | | | |
| Trial design | 3a | Description of trial design (such as parallel, factorial) including allocation ratio | 5-8, supplement 2-3 |
|  | 3b | Important changes to methods after trial commencement (such as eligibility criteria), with reasons | NA |
| Participants | 4a | Eligibility criteria for participants | 5, supplement 2-3 |
|  | 4b | Settings and locations where the data were collected | 5, supplement 2-3 |
| Interventions | 5 | The interventions for each group with sufficient details to allow replication, including how and when they were actually administered | supplement 2-3 |
| Outcomes | 6a | Completely defined pre-specified primary and secondary outcome measures, including how and when they were assessed | NA (exploratory trial) |
|  | 6b | Any changes to trial outcomes after the trial commenced, with reasons | supplement 2-3 |
| Sample size | 7a | How sample size was determined | supplement 2-3 |
|  | 7b | When applicable, explanation of any interim analyses and stopping guidelines | NA |
| Randomisation: |  |  |  |
| Sequence generation | 8a | Method used to generate the random allocation sequence | 5 |
|  | 8b | Type of randomisation; details of any restriction (such as blocking and block size) | 5 |
| Allocation concealment mechanism | 9 | Mechanism used to implement the random allocation sequence (such as sequentially numbered containers), describing any steps taken to conceal the sequence until interventions were assigned | 5 |
| Implementation | 10 | Who generated the random allocation sequence, who enrolled participants, and who assigned participants to interventions | 5 |
| Blinding | 11a | If done, who was blinded after assignment to interventions (for example, participants, care providers, those assessing outcomes) and how | 6 |
|  | 11b | If relevant, description of the similarity of interventions | NA |
| Statistical methods | 12a | Statistical methods used to compare groups for primary and secondary outcomes | 8-9 |
|  | 12b | Methods for additional analyses, such as subgroup analyses and adjusted analyses | 8-9, 11-13 |
| Results | | | |
| Participant flow (a diagram is strongly recommended) | 13a | For each group, the numbers of participants who were randomly assigned, received intended treatment, and were analysed for the primary outcome | 5, 9, Fig.1 |
|  | 13b | For each group, losses and exclusions after randomisation, together with reasons | 9, Fig.1 |
| Recruitment | 14a | Dates defining the periods of recruitment and follow-up | 5-6 |
|  | 14b | Why the trial ended or was stopped | NA |
| Baseline data | 15 | A table showing baseline demographic and clinical characteristics for each group | Table 1 |
| Numbers analysed | 16 | For each group, number of participants (denominator) included in each analysis and whether the analysis was by original assigned groups | Fig. 1 |
| Outcomes and estimation | 17a | For each primary and secondary outcome, results for each group, and the estimated effect size and its precision (such as 95% confidence interval) | 10-13  Table 2,3 |
|  | 17b | For binary outcomes, presentation of both absolute and relative effect sizes is recommended | NA |
| Ancillary analyses | 18 | Results of any other analyses performed, including subgroup analyses and adjusted analyses, distinguishing pre-specified from exploratory | 10-13 |
| Harms | 19 | All important harms or unintended effects in each group (for specific guidance see CONSORT for harms) | NA |
| Discussion | | | |
| Limitations | 20 | Trial limitations, addressing sources of potential bias, imprecision, and, if relevant, multiplicity of analyses | 17-18 |
| Generalisability | 21 | Generalisability (external validity, applicability) of the trial findings | 17-18 |
| Interpretation | 22 | Interpretation consistent with results, balancing benefits and harms, and considering other relevant evidence | 13-18 |
| Other information | | |  |
| Registration | 23 | Registration number and name of trial registry | 5 |
| Protocol | 24 | Where the full trial protocol can be accessed, if available | NA |
| Funding | 25 | Sources of funding and other support (such as supply of drugs), role of funders | 19 |
